# Genome-Wide Identification of Cytokinin Response Factors (CRFs) Involved in Stress Responses in Banana (*Musa acuminata*)

**DOI:** 10.3390/ijms262311316

**Published:** 2025-11-23

**Authors:** Ruiyu Wang, Chunhua Hu, Zhixin Li, Yaoyao Li, Weidi He, Ou Sheng, Qiaosong Yang, Tongxin Dou, Cancan Liu, Huijun Gao, Tao Dong, Ganjun Yi, Shulan Sun, Guiming Deng

**Affiliations:** 1 Institute of Fruit Tree Research, Guangdong Academy of Agricultural Sciences, Ministry of Agriculture and Rural Affairs, Key Laboratory of South Subtropical Fruit Biology and Genetic Resource Utilization, Guangdong Provincial Key Laboratory of Science and Technology Research on Fruit Tree, Guangzhou 510640, Chinaheweidi89@163.com (W.H.);; 2 College of Life Sciences, South China Normal University, Guangzhou 510640, China; 3 College of Horticulture and Forestry Sciences, Huazhong Agricultural University, Wuhan 430070, China

**Keywords:** banana, cytokinin response factor (*CRF*), biotic stress, abiotic stress, expression analysis

## Abstract

Cytokinin response factors (CRFs), belonging to AP2/ERF transcription factor family, play pivotal roles in regulating plant growth, hormone signaling, and stress responses. While CRF genes have been functionally characterized in multiple plant species, their systematic analysis in banana (*Musa* spp.), a globally important tropical fruit crop, remains unexplored. In this study, we identified eight putative MaCRF genes in the wild banana *Musa acuminata* ssp. malaccensis var. Pahang. Through comprehensive bioinformatic analyses, we characterized the MaCRF family and investigated their expression profiles across diverse tissues and under various biotic and abiotic stresses. Intriguingly, *MaCRF4* exhibited contrasting expression patterns in response to *Fusarium oxysporum* f. sp. cubense tropical race 4 (*Foc* TR4) infection. *MaCRF4* was strongly induced in the susceptible cultivar Z1 but transiently upregulated at early stages followed by downregulation in the resistant cultivar Z8. Furthermore, *MaCRF3* and *MaCRF4* were markedly induced by osmotic stress, low temperature, salinity, and ABA treatment. Our findings provide the systematic characterization of the MaCRF family in banana and offer valuable insights for future functional studies aimed at enhancing stress tolerance through molecular breeding strategies.

## 1. Introduction

The transcription factor superfamily of AP2/ERF (APETALA2/ethylene-responsive factor) is widely distributed across the plant kingdom. A defining feature of this superfamily is the AP2 domain, as a conserved DNA-binding motif consisting of 60–70 amino acids [1,2]. Among these transcription factors, the cytokinin response factors (CRFs) constitute a specialized clade of ERF-type members [2]. Structurally, CRF proteins typically contain a single AP2/ERF domain, a highly conserved CRF-specific domain, and a variable C-terminal domain, which facilitates protein–protein interactions and distinguishes CRFs from other AP2/ERF transcription factors [3,4]. To date, CRF genes have been identified and functionally characterized in only a limited number of plant species, including 12 members in Arabidopsis (*Arabidopsis thaliana*) [2,3], 11 members in tomato (*Solanum lycopersicum*) [5], 21 members in Chinese cabbage (*Brassica oleracea* var. *capitata*) [6], 44 members in rapeseed (*Brassica napus*) [7], 26 members in legumes (*Glycine max*) [8], 7 members in rice (*Oryza sativa*) [9], and 12 members in maize (*Zea mays*) [10]. Despite these advances, CRF genes remain poorly characterized in many other species, and their precise biological functions are still not fully elucidated.

Extensive research across various plant species has revealed the multifaceted roles of CRF genes in growth and developmental processes, mediating hormone signaling pathways, and coordinating responses to diverse biotic and abiotic stresses [11,12,13]. In Arabidopsis, CRF2 confers broad-spectrum pathogen resistance through direct transcriptional activation of defense-related genes, binding specifically to the promoters of PR1 and PDF1.2 to enhance resistance against *Pseudomonas syringae* and *Botrytis cinerea* [14]. *AdCRF6* mediates systemic acquired resistance by regulating the biosynthesis of methionine-derived mobile signaling molecules that activate defense responses in distal tissues [15]. *SlCRF1* exhibits specialized function in nematode defense, potentiating JA/ET signaling pathways to combat Meloidogyne incognita infection [5]. *GmCRF4a* coordinates root architectural plasticity during pathogen challenge by fine-tuning auxin biosynthesis, maintaining optimal hormone homeostasis for defense responses [16]. *GmCRF3* and *GmCRF15* show tissue-specific cold induction profiles in soybean [16]. *OsCRF1* exhibits multi-stimuli responsiveness to cold, melatonin and cytokinin in rice [9]. *ZmCRF9* serves as a positive regulator of both cold and salt stress tolerance in maize [10]. Additionally, in rapeseed, certain *CRF* genes in rapeseed contribute to enhanced tolerance to low-phosphorus conditions [7].

Banana (*Musa* spp.), a crucial tropical fruit crop, holds significant global agricultural importance as the world’s fourth most produced food commodity after rice, wheat, and maize [17,18]. However, banana production faces severe challenges from Fusarium wilt, a devastating soil-borne disease caused by *Fusarium oxysporum* f. sp. *cubense* (*Foc*) [19,20,21]. Among the various pathogenic forms, *Foc* tropical race 4 (*Foc* TR4) is the most destructive, particularly in China. *Foc* TR4 infect *Cavendish* bananas and cultivars susceptible to race 1, 2, and subtropical race 4 [22,23,24,25]. This crisis underscores the urgent need for international collaboration to safeguard banana production systems against this expanding biological threat [22,23,24,25].

Banana cultivation demonstrates exceptional vulnerability to various environmental stressors, with production being substantially compromised by extreme temperature fluctuations (both high and low), water-related stresses (drought and excessive rainfall), soil salinity, and severe wind conditions. While the AP2/ERF superfamily of transcription factors has been well documented in banana genomes [26], the CRF subfamily remains completely unexplored. Notably, the potential involvement of CRF genes in mediating banana’s responses to both biotic and abiotic stress conditions has yet to be elucidated.

To address this critical knowledge gap, we conducted a systematic genome-wide investigation of the CRF gene family using the sequenced banana subspecies *Musa acuminata* (DH Pahang) [17]. We performed a comprehensive characterization of MaCRF genes through multi-dimensional analyses. The analysis focused on gene and protein features, including physicochemical properties, phylogenetic relationships, conserved motifs, gene structures, chromosomal distribution, duplication events, cis-acting elements, and expression patterns. To functionally contextualize these findings, we conducted comparative expression profiling in two phenotypically distinct banana cultivars, the Fusarium wilt-susceptible Zhongjiao 1 and resistant Zhongjiao 8. Transcriptional responses to both *Foc* TR4 infection and an array of abiotic stressors including thermal, osmotic, and ionic challenges were conducted. Our integrated analysis not only elucidates the molecular architecture of banana CRF genes, but also reveals their stress-responsive expression patterns, offering a foundation for future functional studies.

## 2. Results

### 2.1. Identification and Characterization of MaCRF Family Genes in M. acuminata DH Pahang

To systematically identify CRF genes in the *M. acuminata* DH Pahang genome, we employed a comprehensive bioinformatics approach. A local BLAST database was established with TBtools-II software using known CRF protein sequences from *A. thaliana* and *O. sativa* as reference queries was established (Table S2). Candidate sequences were then rigorously filtered through Pfam domain analysis (PF00847) to verify the presence of complete AP2/ERF domains and ensure the structural integrity. Through this stringent screening process, eight putative CRF genes, designated MaCRF1-8 in the *M. acuminata* genome were identified. These genes exhibited considerable variation in their coding sequences, ranging from 576 bp (*MaCRF3*) to 978 bp (*MaCRF8*), corresponding to proteins of 191 to 326 amino acids.

Bioinformatic characterization revealed that the encoded proteins display diverse physicochemical properties. Molecular weights ranging from 21.36 kDa (*MaCRF3*) to 35.68 kDa (*MaCRF8*). The theoretical isoelectric points spanned from acidic (5.22 for *MaCRF2*) to basic (10.27 for *MaCRF3*), and the instability indices between 54.24 and 69.21, classified them as unstable proteins. In addition, the grand average of hydropathy (GRAVY) values ranged from −1.294 to −0.296, indicating them with hydrophilic character. Subcellular localization predictions suggested nuclear targeting for most MaCRF proteins, with some showing potential chloroplast localization (Table S1). These findings provide fundamental insights into the structural characteristics of the CRF gene family in banana.

### 2.2. Phylogenetic Analysis and Multiple Sequence Alignment of Banana CRF Family Proteins

**Figure 1:**
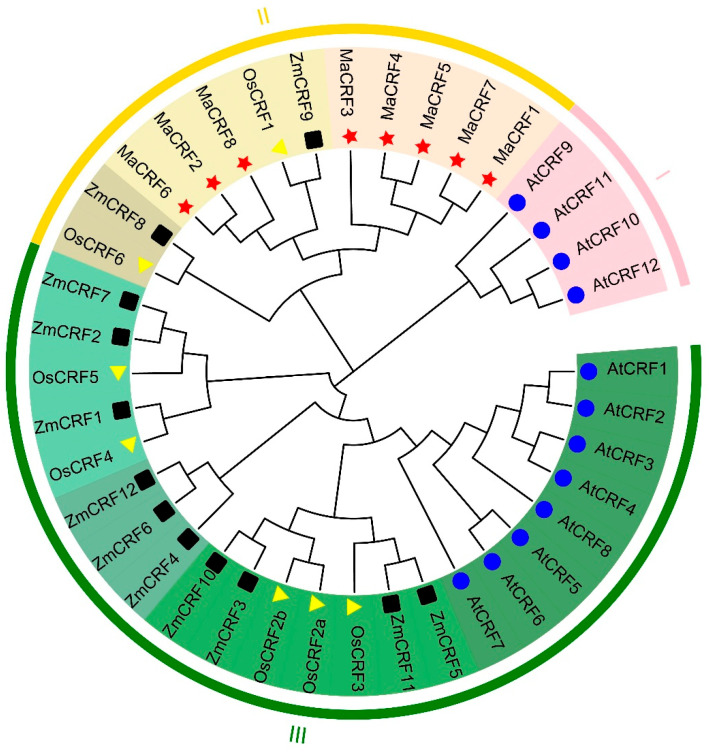
Phylogenetic tree of CRF proteins from *M. acuminata* DH Pahang, *A. thaliana*, *O. sativa*, and *Z. mays*. The phylogenetic tree was constructed using MEGA X software with the Neighbor joining (NJ) method based on multiple sequence alignment of CRF proteins s from *M. acuminata* DH Pahang (8), *A. thaliana* (12), *O. sativa* (7), and *Z. mays* (12). The CRF proteins were divided into three groups based on phylogenetic clustering, each represented by a distinct color. Species-specific markers are indicated as follows: blue circles for *A. thaliana*, dark squares for *Z. mays*, light yellow triangles for *O. sativa*, and red pentagram for *M. acuminata* DH Pahang. Abbreviations: At, *A. thaliana*; Ma, *M. acuminata*; Os, *O. sativa*; Zm, *Z. mays*.

To elucidate the evolutionary relationships among CRF proteins, phylogenetic analysis using representative sequences from *A. thaliana* (Arabidopsis), *O. sativa* (rice), *Z. mays* (maize), and *M. acuminata* was performed (Figure 1). The analysis of 39 CRF proteins revealed three distinct subfamilies (I–III), with all eight banana CRF proteins exclusively clustering within Subfamily II. This subfamily included *OsCRF1*, *OsCRF6*, *ZmCRF8*, and *ZmCRF9*, suggesting conserved evolutionary patterns among monocots.

The banana CRF proteins formed two well-defined clades (color-coded in Figure 1), with members within each clade showing high structural homology, indicative of potential functional conservation. Comparative analysis revealed significant variation in CRF family size across species, reflecting substantial evolutionary divergence. CRF proteins in banana demonstrated closer phylogenetic relationships to their monocot counterparts (rice and maize) than to Arabidopsis CRFs, supporting the expected evolutionary divergence between monocots and dicots.

### 2.3. Gene Structures, and Motifs Analysis of Banana CRF Family Proteins

Gene structure analysis revealed distinct architectural features among the eight MaCRF genes (Figure S1 and S2). Notably, among these genes, *MaCRF7* represents an intronless gene, while six members (*MaCRF3-8*) exhibit a single-exon structure. In contrast, *MaCRF1* and *MaCRF2* contain more complex gene structures with three and two exons, respectively. UTR analysis showed additional variability, where *MaCRF1* and *MaCRF2* completely lack UTRs, *MaCRF7* contains a single UTR, while the remaining genes possess three UTRs each.

Conserved protein motif analysis identified ten characteristic motifs (Figure S1), with all MaCRF proteins sharing Motifs 1 and 3, indicating a conserved functional core. The variable presence and arrangement of other motifs among the family members suggest structural diversification that may underlie functional specialization within the *MaCRF* family.

### 2.4. Comprehensive Analysis of Cis-Regulatory Elements in MaCRF Promoters

**Figure 2:**
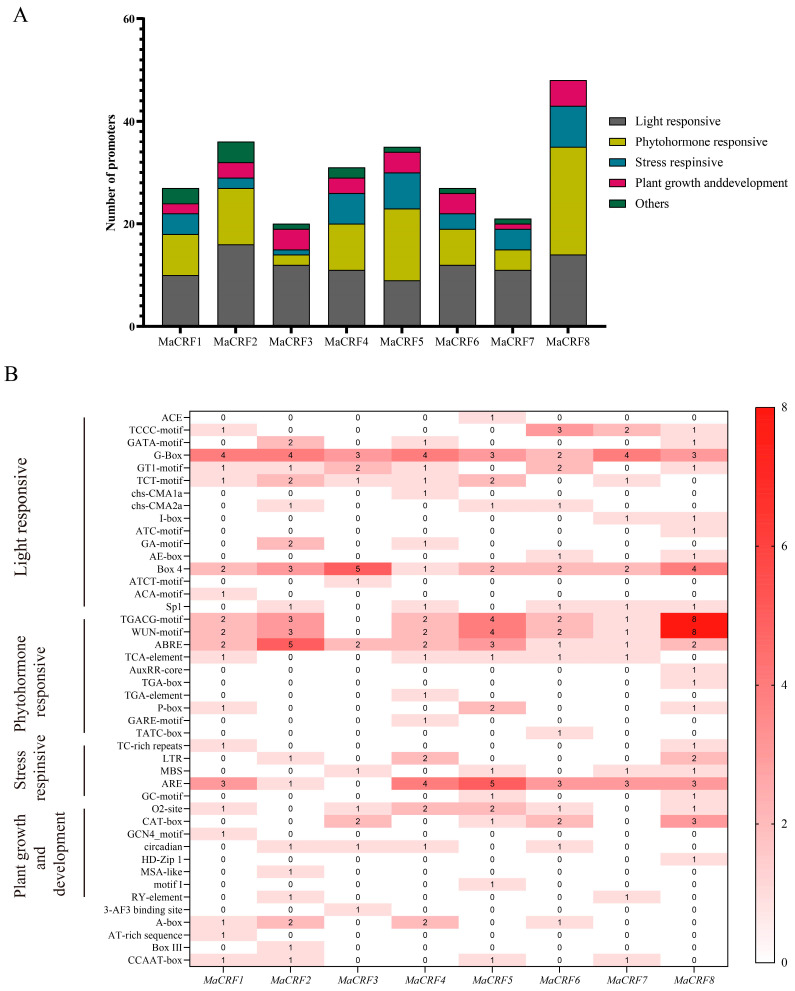
Prediction of cis-acting elements in the promoters of *M. acuminata* DH Pahang *CRF* family genes. Cis-acting elements within the 2-kb upstream promoter regions of MaCRF genes were predicted using the PlantCARE database. (**A**) Stacked plot of cis-acting elements. Different colors represent different types of cis-acting elements. (**B**) Cis-acting element heatmap. Colored boxes represent different categories of cis-acting elements, with varying shades of red indicating the relative abundance of each element type. The darker the red, the greater the number of corresponding cis-elements.

Cis-acting elements in the promoter regions of *MaCRF* genes were predicted using the PlantCARE database. Bioinformatic analysis of the 2-kb promoter regions upstream of MaCRF genes revealed an abundance of diverse cis-acting regulatory elements, which we classified into three major functional categories (Figure 2 and Table S3). Specifically, numerous light-responsive elements were identified, including ACE, TCCC-motif, GATA-motif, G-Box, GT1-motif, TCT-motif, chs-CMA1a, chs-CMA2a, I-box, ATC-motif, GA-motif, AE-box, Box 4, ATCT-motif, ACA-motif. Hormone-responsive elements included TGACG-motif, WUN-motif, ABRE, TCA-element, AuxRR-core, TGA-box, TGA-element, P-box, GARE-motif, TATC-box, and Sp1. Additionally, stress-related elements such as TC-rich repeats, LTR, MBS, ARE, GC-motif, as well as plant growth and development elements such as O_2_-site, CAT-box, GCN4_motif, circadian, HD-Zip 1, MSA-like, motif I, RY-element were identified (Table S3). These findings strongly suggest that MaCRF genes play multifaceted roles in banana growth and development, while serving as key integrators of hormone and environmental stress responses.

### 2.5. Chromosomal Localization and Collinearity Analysis of Banana CRF Family Genes

**Figure 3:**
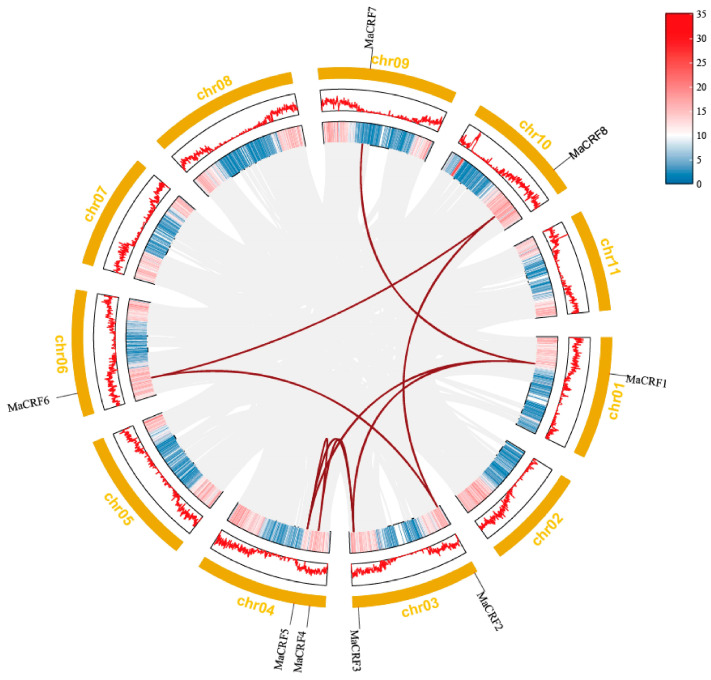
Chromosomal localization and collinearity analysis of banana CRF family genes in *M. acuminata* DH Pahang. Eight *MaCRF* genes were mapped on five chromosomes of *M. acuminata* DH Pahang genome. Gene duplication events were identified using the MCScanX program in TBtools software and are illustrated by red connecting lines between duplicated gene pairs. Gray lines represent all the collinear blocks within the genomes. The inner circle represents gene density, while the outer yellow ring denotes chromosomes.

Chromosomal mapping analysis demonstrated that the eight *MaCRF* genes are distributed across six chromosomes in banana (Figure 3). Specifically, *MaCRF1* was located on chromosome 1, while chromosomes 3, 4, 6, 9 and 10 harbored *MaCRF2/3*, *MaCRF4/5*, *MaCRF6*, *MaCRF7* and *MaCRF8*, respectively. Gene duplication analysis identified eight segmental duplication events involving all MaCRF members (*MaCRF1*–*MaCRF3*, *MaCRF1*–*MaCRF5*, *MaCRF1*–*MaCRF7*, *MaCRF2*–*MaCRF6*, *MaCRF2*–*MaCRF8*, *MaCRF3*–*MaCRF5*, *MaCRF4*–*MaCRF5*, *MaCRF6*–*MaCRF8*), suggesting that whole-genome or segmental duplications have played a major role in the expansion of the CRF gene family in banana. We calculated the Ka, Ks, and Ka/Ks values of these 8 gene pairs using the Simple Ka/Ks Calculator (NG) in TBtools. The results showed that all Ka/Ks values were less than 1, indicating that the gene sequences are more conserved (Table S5). Furthermore, interspecies collinearity analysis detected only one conserved syntenic gene pair between banana and rice (located on chromosome 10; Figure S2), implying that this CRF gene may have maintained important conserved functions during the evolution of these two divergent monocot species.

### 2.6. Secondary Structure Prediction of Banana CRF Family Proteins

Secondary structure analysis of banana CRF proteins was performed using the SOPMA online tool (Table 1). The eight MaCRF proteins exhibited four characteristic structural elements including α-helices (15.64–31.96%), extended strands (4.12–8.45%), β-turns (1.23–2.62%), and random coils (61.86–78.22%). Notably, α-helices and random coils constituted the major structural components across all proteins, with MaCRF1 showing the highest α-helix content (31.96%) and MaCRF8 containing the most random coils (78.22%). This structural composition suggests that banana CRF proteins possess considerable conformational flexibility, which may be important for their functional diversity.

### 2.7. Tissue-Specific Expression Analysis of Banana CRF Family Genes

**Figure 4:**
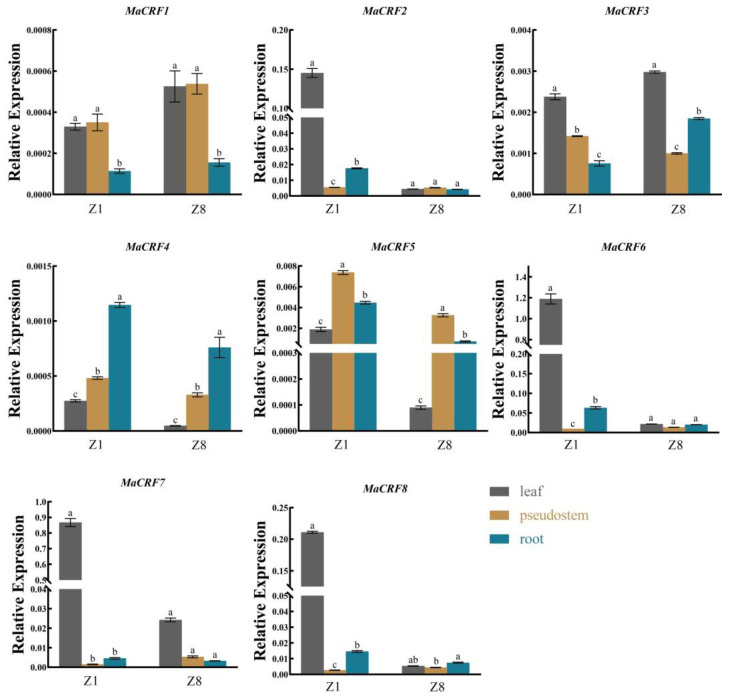
Tissue-specific expression profiles of *MaCRF* genes in *M. acuminata* DH Pahang. Expression levels of *MaCRF* genes were analyzed in different tissues (leaves, pseudostems, and roots) across of two banana cultivars (Zhongjiao 1, Z1; and Zhongjiao 8, Z8). Data are presented as mean ± standard deviation (S.D.) from three biological replicates. Statistical significance was determined using Tukey’s HSD test. In the same species, the presence of at least one identical letter indicates no significant difference, while the absence of any identical letters denotes a significant difference.

To investigate the functional diversification of MaCRF genes, comparative expression analysis in leaves, pseudostems and roots of two banana cultivars (Zhongjiao 1, Z1; Zhongjiao 8, Z8, Figure 4). Our results revealed distinct spatiotemporal expression patterns, *MaCRF6* and *MaCRF7* showed predominant expression in Z1 leaves, while *MaCRF2* and *MaCRF8* showed elevated expression in Z1 leaves compared to those of cultivar Z8. In contrast, *MaCRF1*, *MaCRF3 MaCRF4* and *MaCRF5* displayed relatively low expression levels across all examined tissues.

In contrast, *MaCRF1*, *MaCRF3 MaCRF4* and *MaCRF5* displayed relatively low expression levels across all examined tissues. The differential regulation of MaCRF genes across tissues and cultivars strongly supports functional diversification within this transcription factor family, likely reflecting their adaptation to various developmental and environmental cues in banana.

### 2.8. Expression Profiles of Banana CRF Family Genes Under Biotic Stress

**Figure 5:**
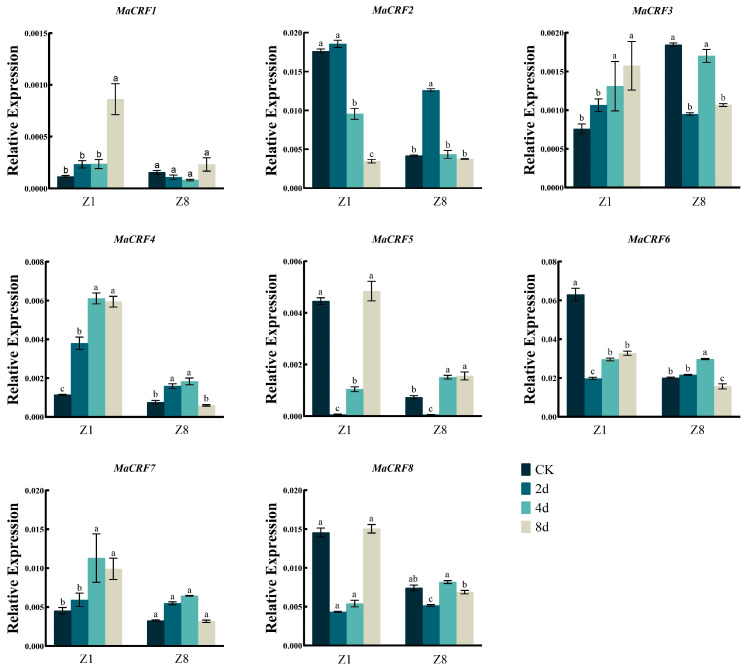
qRT-PCR analysis of *MaCRF* gene expression in two banana cultivars under *Foc* TR4 infection. Expression profiles of MaCRF genes were examined in two banana cultivars—Zhongjiao 1 (Z1, susceptible to *Fusarium oxysporum* f. sp. cubense tropical race 4, *Foc* TR4) and Zhongjiao 8 (Z8, resistant to *Foc* TR4)—at multiple time points following inoculation. CK, control; 2d, 2 days post-inoculation; 4d, 4 days post-inoculation; 8d, 8 days post-inoculation. Data are presented as mean ± standard deviation (S.D.) from three biological replicates. Statistical significance was determined using Tukey’s HSD test. In the same species, the presence of at least one identical letter indicates no significant difference, while the absence of any identical letters denotes a significant difference.

The expression dynamics of eight *MaCRF* genes were analyzed in two banana Zhongjiao 1 (Z1, susceptible to *Fusarium oxysporum* f. sp. *cubense* tropical race 4, *Foc* TR4) and Zhongjiao 8 (Z8, resistant to *Foc* TR4) at multiple time points following *Foc* TR4 inoculation (Figure 5). Distinct expression patterns were observed between cultivars. After infection *Foc* TR4, *MaCRF1* and *MaCRF7* were significantly upregulated in Z1, while their expression remained stable in Z8. *MaCRF2* and *MaCRF6* were down regulated in Z1, but exhibited a transient increase and decline immediately in Z8. *MaCRF3* exhibited opposing trends, being upregulated in Z1 and downregulated in Z8. *MaCRF4* showed strong induction in Z1, but in Z8 it was transiently upregulated during the early stage of infection, followed by gradual downregulation. *MaCRF5* and *MaCRF8* were downregulated at early stages in both varieties. These contrasting expression patterns suggest that the differential regulation of *MaCRF* genes may be associated with the distinct response of resistance to *Foc* TR4 between the two cultivars.

### 2.9. Expression Responses of MaCRF Genes to Abiotic Stresses

**Figure 6:**
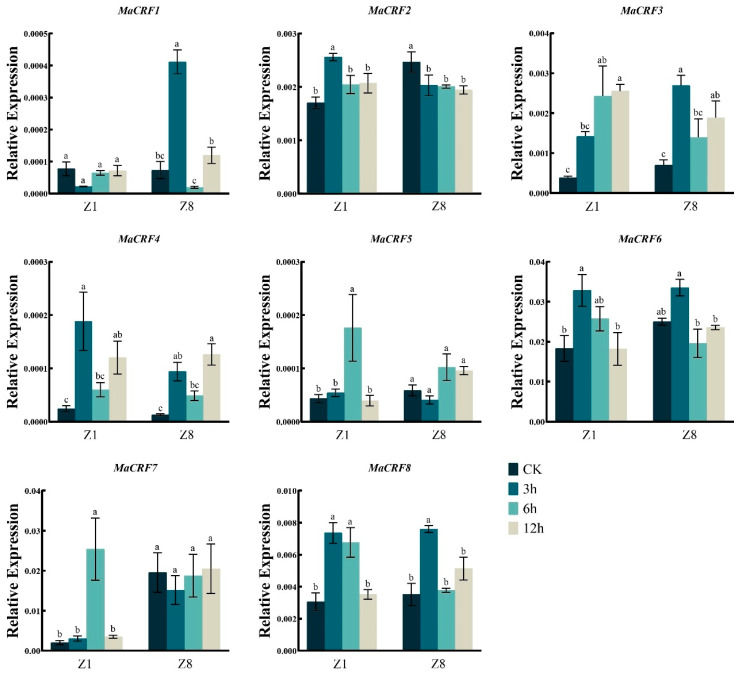
Expression analysis of *MaCRF* genes under PEG6000 stress in two banana cultivars. The expression profiles of *MaCRF* genes were evaluated in Zhongjiao 1 (Z1) and Zhongjiao 8 (Z8) under PEG6000 stress. Color gradients represent relative expression levels at different time points. PEG6000, simulated drought treatment. CK, control; 3h, 3 hours; 6h, 6 hours; 12h, 12 hours. Data are presented as mean ± standard deviation (S.D.) from three biological replicates. Statistical significance was determined using Tukey’s HSD test. In the same species, the presence of at least one identical letter indicates no significant difference, while the absence of any identical letters denotes a significant difference.

**Figure 7:**
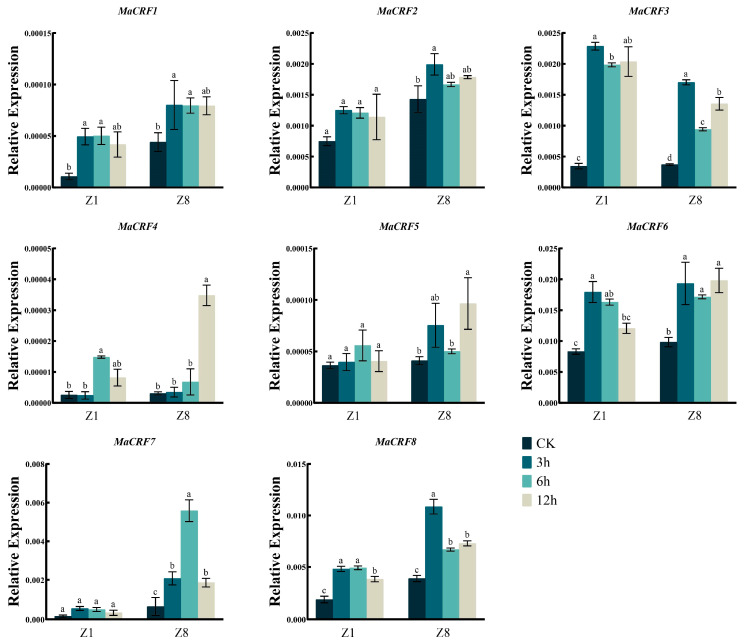
Expression analysis of *MaCRF* genes under NaCl stress in two banana cultivars. The expression profiles of *MaCRF* genes were evaluated in Zhongjiao 1 (Z1) and Zhongjiao 8 (Z8) under NaCl stress. Color gradients represent relative expression levels at different time points. CK, control; 3h, 3 hours; 6h, 6 hours; 12h, 12 hours. Data are presented as mean ± standard deviation (S.D.) from three biological replicates. Statistical significance was determined using Tukey’s HSD test. In the same species, the presence of at least one identical letter indicates no significant difference, while the absence of any identical letters denotes a significant difference.

**Figure 8:**
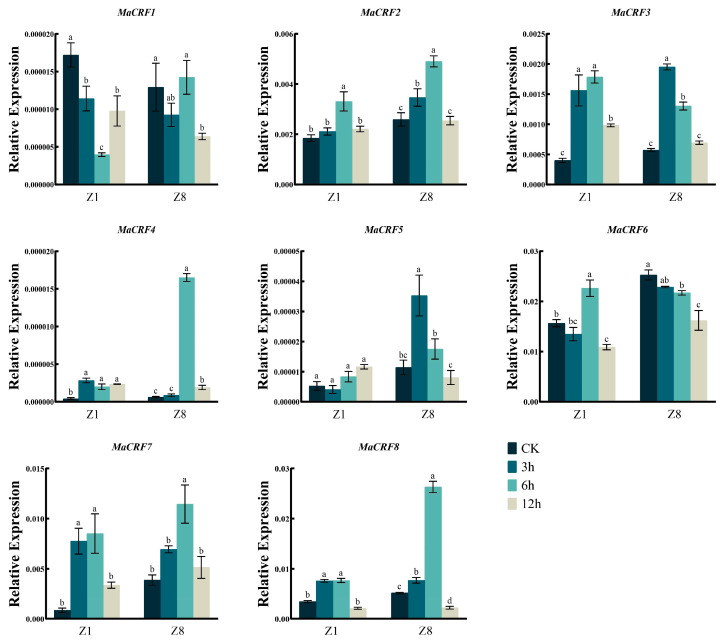
Expression analysis of *MaCRF* genes under ABA stress in two banana cultivars. The expression profiles of *MaCRF* genes were evaluated in Zhongjiao 1 (Z1) and Zhongjiao 8 (Z8) under ABA stress. Color gradients represent relative expression levels at different time points. CK, control; 3h, 3 hours; 6h, 6 hours; 12h, 12 hours. Data are presented as mean ± standard deviation (S.D.) from three biological replicates. Statistical significance was determined using Tukey’s HSD test. In the same species, the presence of at least one identical letter indicates no significant difference, while the absence of any identical letters denotes a significant difference.

**Figure 9:**
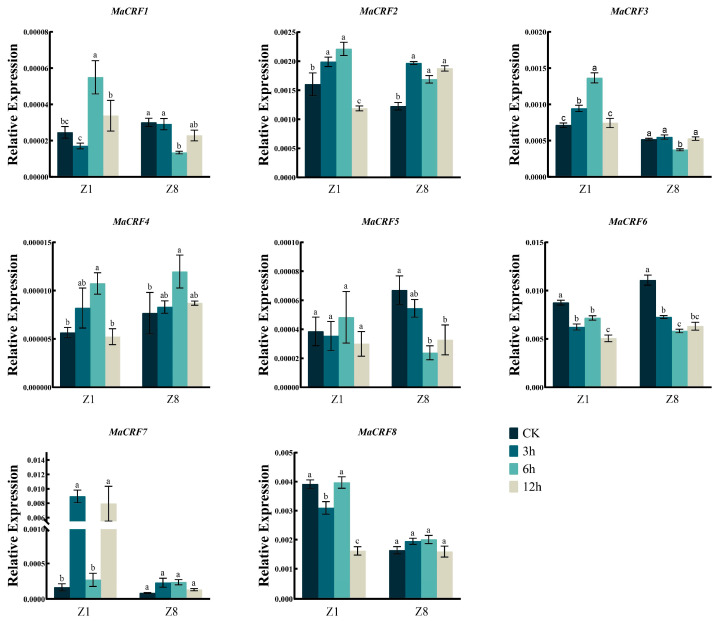
Expression analysis of *MaCRF* genes under 4 °C stress in two banana cultivars. The expression profiles of *MaCRF* genes were evaluated in Zhongjiao 1 (Z1) and Zhongjiao 8 (Z8) under 4 °C stress. Color gradients represent relative expression levels at different time points. CK, control; 3h, 3 hours; 6h, 6 hours; 12h, 12 hours. Data are presented as mean ± standard deviation (S.D.) from three biological replicates. Statistical significance was determined using Tukey’s HSD test. In the same species, the presence of at least one identical letter indicates no significant difference, while the absence of any identical letters denotes a significant difference.

We further examined the expression profiles of MaCRF family genes under various abiotic stresses, including osmotic stress (PEG6000), salt (NaCl), abscisic acid (ABA), and low-temperature stress. Generally, the eight MaCRF genes exhibited distinct, stress- and genotype-dependent expression patterns (Figure 6, Figure 7, Figure 8 and Figure 9).

Under PEG6000-induced osmotic stress, *MaCRF1* was significantly upregulated in the resistant cultivar Z8 but remained stable in the susceptible Z1 (Figure 6). Conversely, *MaCRF5* and *MaCRF7* were strongly induced in Z1 but showed no significant change in Z8. Notably, *MaCRF2* was upregulated in Z1 but downregulated in Z8, while *MaCRF3*, *MaCRF4*, *MaCRF6*, and *MaCRF8* were downregulated at early stages in both cultivars. These results suggest that MaCRF genes are differentially regulated in a genotype-specific manner under osmotic stress.

*MaCRF1*, *MaCRF3*, *MaCRF4*, *MaCRF6*, and *MaCRF8* were upregulated at the early stage in both Z1 and Z8 after NaCl treatment (Figure 7). *MaCRF2* and *MaCRF5* were specifically induced during the early stage of salt stress in Z8, without obvious changes observed in Z1. *MaCRF7* expression remained unchanged in Z1 but was transiently upregulated in Z8 during the early stage, followed by downregulation at later time points (Figure 7). These findings highlight differential and genotype-specific regulatory responses of *MaCRF* genes to salt stress.

After ABA treatment, *MaCRF2*, *MaCRF3*, *MaCRF4*, *MaCRF7*, and *MaCRF8* were upregulated at the early stage of treatment in both Z1 and Z8, whereas *MaCRF1* and *MaCRF6* were downregulated in both varieties (Figure 8). Notably, *MaCRF4* exhibited a significant induced at 6 h in Z8, while its expression remained changed slightly in Z1. In contrast, *MaCRF5* was downregulated in Z1 during the early response but significantly upregulated in Z8 (Figure 8). These results demonstrate that ABA-mediated stress responses involve genotype-dependent regulation of *MaCRF* genes. In addition, *MaCRF1*, *MaCRF2*, *MaCRF4*, *MaCRF5*, and *MaCRF6* displayed similar expression patterns during the early stage of cold treatment in both Z1 and Z8 (Figure 9). *MaCRF3* and *MaCRF7* were induced in Z1, while *MaCRF8* was downregulated in Z1 but changed less in Z8. These observations underscore the divergent and genotype-specific regulatory roles of MaCRF genes in cold stress responses. Collectively, these findings reveal that MaCRF genes exhibit dynamic, stress-specific, and cultivar-dependent expression patterns, suggesting their potential roles in mediating differential stress adaptation between the susceptible (Z1) and resistant (Z8) banana cultivars.

## 3. Discussion

The AP2/ERF family, one of the largest transcription factor families in plants, plays crucial roles in growth, development, abiotic stress responses, and signal transduction [9]. Among its subfamilies, CRFs have been extensively studied in *Arabidopsis thaliana* and, to a lesser extent, in other species such as tomato, cabbage, soybean, Tamarix chinensis, Quercus variabilis, rice, and maize [5,9,10,27,28,29,30]. In this study, we systematically identified AP2/ERF members in banana and screened for CRF-type transcription factors through multiple sequence alignments with *A. thaliana* CRFs. A total of eight CRF genes were identified in banana, consistent with the variable number of CRF genes observed across plant species, which does not strictly correlate with genome size. For instance, *A. thaliana* contains 12 CRF genes [3], tomato 11 [5], soybean 26 [8], rice 7 [9], and maize 12 [10]. Physicochemical property analysis revealed that all banana CRF proteins are hydrophilic and unstable, with subcellular localization predictions indicating predominant nuclear localization—features typical of transcription factors and consistent with findings in maize [10]. Phylogenetic analysis demonstrated significant interspecific divergence among CRF proteins from *A. thaliana*, rice, maize, and banana. Notably, banana CRFs clustered more closely with those of rice and maize, suggesting a conserved evolutionary relationship within monocots. Promoter analysis identified cis-acting elements associated with hormone responses, growth and development, and abiotic stress responses in banana CRF genes, aligning with previous reports in soybean [8]. Secondary structure predictions revealed that banana CRF proteins are predominantly composed of α-helices (19.98%) and random coils (72.48%), a structural composition similar to that of rice CRFs [9]. Together, these findings provide valuable insights into the evolutionary history and potential functional conservation of CRF proteins in monocots.

Tissue-specific expression analysis revealed differential expression of *MaCRF* genes across banana tissues, suggesting their involvement in diverse developmental processes. The spatial regulation implies specialized biological functions for individual *MaCRF* members during banana growth and development. Given the devastating impact of Fusarium wilt (caused by *Fusarium oxysporum* f. sp. *cubense* tropical race 4, *Foc* TR4) on global banana production [31], *MaCRF* transcriptional responses in resistant (Z8) and susceptible (Z1) cultivars were tested. Notably, *MaCRF4* showed strong induction in susceptible Z1 under *Foc* TR4 infection, while in resistant Z8 it exhibited transient early upregulation followed by gradual downregulation, suggesting a potential role in root cell defense mechanisms. Generally MaCRF expression patterns maintained more stable in Z8 compared to Z1, indicating possible cultivar-specific defense regulation. These findings align with results in Arabidopsis, where *AtCRF2* and *AtCRF5* overexpression enhanced resistance to *Pseudomonas syringae* accompanied with the upregulation of pathogenesis-related genes [14,32].

Promoter analyses identified stress-related and ABA-responsive cis-elements in MaCRF genes, consistent with their roles in stress adaptation. Most of the MaCRF genes were downregulated under PEG6000 treatment, being consistent with CRF expression profiles reported in soybean [33]. *MaCRF1*, *MaCRF3*, *MaCRF4*, *MaCRF6*, and *MaCRF8* were significantly upregulated in both cultivars induced by salt stress, which has been similarly reported for *ThCRF1* in *Tamarix hispida* [34]. MaCRF genes exhibited upregulation in both cultivars following ABA treatment, exhibited potential roles in ABA-mediated growth regulation, while its regulation mechanisms are still uncertain [35].

In addition, banana is susceptible to the environmental temperature, especially chilling injury for both plants and fruits under low temperature stress [36]. CRFs also play important roles in response to the cold stress in plant. For instance, *AtCRF2* and *AtCRF3* can promote the initiation and formation of lateral roots under low temperature, and the expression of *CRF2* is directly regulated by ARR1 [37]. In soybean, nearly all CRF genes are transcriptionally induced by cold treatment [33]. In banana, *MaCRF2* was upregulated in both Z1 and Z8 cultivars, mirroring cold-responsive *ZmCRF9* in maize [10]. *MaCRF3* and *MaCRF4* showed cultivar-specific induction in Z1. MaCRF3 and MaCRF7 were induced in Z1, while MaCRF8 was downregulated in Z1 but changed less in Z8. These observations underscore the divergent and genotype-specific regulatory roles of MaCRF genes in cold stress responses. Collectively, these findings reveal that MaCRF genes exhibit dynamic, stress-specific, and cultivar-dependent expression patterns, suggesting their potential roles in mediating differential stress adaptation between the susceptible (Z1) and resistant (Z8) banana cultivars.

## 4. Materials and Methods

### 4.1. Identification of CRF Family Members and Analysis of Protein Physicochemical Properties

To analysis the CRF family members, genome wide identification was performed. Genome data source was searched as the reference genome of *Musa acuminata* (DH Pahang) obtained from the Banana Genome Hub database (https://banana-genome-hub.southgreen.fr/data_search/organism, accessed on 29 October 2025) [38]. AP2 domain HMM profile retrieval was done by the Hidden Markov Model (HMM) profile for the AP2 domain (PF00847) acquired from the Pfam database. Candidate AP2 family genes were initially screened using TBtools-II [39], employing the AP2 domain HMM (PF00847) to conduct a genome-wide search. To specifically identify CRF family members, through employing default parameters and a significant e^−3^ value, BLASTP searches were performed in TBtools-II using protein sequences of 12 known CRF genes from *Arabidopsis thaliana* and 7 from *Oryza sativa* (rice) [9]. The presence of the AP2 domain in the candidate MaCRF protein sequences was further confirmed using the Conserved Domain Database (CDD) from the National Center for Biotechnology Information (NCBI). Then, the physicochemical properties of the identified MaCRF proteins were evaluated using the ExPASy ProtParam tool (https://web.expasy.org/protparam/, accessed on 29 October 2025) [40]. The subcellular localization of MaCRF proteins was predicted using the WoLF PSORT tool (https://wolfpsort.hgc.jp/, accessed on 29 October 2025) [41].

### 4.2. Phylogenetic Analysis of Banana CRF Family Proteins

Retrieval of CRF protein sequences was performed by searching CRF protein sequences from *Oryza sativa* (rice), *Arabidopsis thaliana* (Arabidopsis), and *Zea mays* (maize) obtained from the EnsemblPlants genome database [42]. A phylogenetic tree was constructed using the Neighbor-Joining method in MEGA X software with default parameter settings [43]. The resulting tree was visualized and refined using the Evolview online tool (http://www.evolgenius.info/evolview/#/treeview, accessed on 29 October 2025) [44].

### 4.3. Analysis of Cis-Acting Elements in the Promoters of Banana CRF Genes

The 2-kb promoter regions upstream of the start codons of MaCRF genes were extracted using TBtools-II [39]. Putative cis-acting regulatory elements were identified through computational prediction using the PlantCARE database (PlantCARE, a database of plant promoters and their cis-acting regulatory elements, accessed on 29 October 2025) [45]. The distribution and functional classification of these elements were subsequently analyzed and visualized using TBtools-II.

### 4.4. Chromosomal Localization and Collinearity Analysis of Banana CRF Genes

*MaCRF* genes positions in the genomic were mapped according to the reference genome annotation, and their chromosomal distribution was visualized using TBtools-II [39]. To investigate evolutionary relationships, collinearity analysis using TBtools-II to identify segmental duplication events and examine syntenic conservation among MaCRF family members were performed.

### 4.5. Plant Materials and Treatments

Plants of Zhongjiao 1 and Zhongjiao 8 (*Musa acuminate* L., genome AAA) were obtained from the Guangzhou Branch of the National Banana Improvement Center (Banana Improvement Center, Institute of Pomology, Guangdong Academy of Agricultural Sciences, Guangzhou, China) and propagated in vitro from banana sucker tissues. The plants were grown until the fifth leaf was fully expanded under conditions of approximately 28–30 °C with a light/dark cycle of about 14/10 h, and then used for inoculation experiments.

The *Fusarium oxysporum* f. sp. cubense tropical race 4 (*Foc* TR4, VCG 01213/16) strain was provided by the Guangzhou Branch of the National Banana Improvement Center. *Foc* TR4 was cultured in potato dextrose broth at 28 °C with shaking at 200 rpm for 48 h. Mycelia were removed by filtration through four layers of sterile gauze, and the spore concentration was counted. The plant roots were immersed in Hoagland nutrient solution containing 10^6^ CFU·g^−1^ *Foc* TR4 in a light incubator under conditions of 28–30 °C with a 14/10 h light/dark cycle. Root samples were collected at 0 days (control), 2 days, 4 days, and 8 days post-inoculation (dpi).

For abiotic stress treatments, banana seedlings were cultured in Hoagland nutrient solution in an artificial climate chamber for two weeks. Subsequently, plants were exposed to the following conditions: 20% PEG6000 (simulated drought), 250 mmol·L^−1^ NaCl (salt stress), 100 µmol·L^−1^ abscisic acid (ABA), or 4 °C (cold stress). Leaf samples were harvested at 0, 3, 6, and 12 h after treatment initiation.

### 4.6. Expression Analysis of Banana CRF Genes

Total RNA was extracted using a commercial RNA extraction kit (Promega, Madison, WI, USA), and first-strand cDNA was synthesized using the FastKing gDNA Dispelling RT SuperMix kit (Tiangen, Beijing, China). Gene-specific primers were designed using Primer-BLAST (NCBI), and primer sequences are provided in Table S4. Quantitative real-time PCR (qRT-PCR) was performed in a 20 μL reaction system containing 10 μL 2 × Tap Pro Universal SYBR qPCR Master Mix (Vazyme, Nanjing, China), 3 μL of cDNA template, 0.4 μL each of forward and reverse primer, and 6.2 μL of nuclease-free water. The amplification protocol was as follows: initial denaturation at 95 °C for 30 s; followed by 40 cycles of 95 °C for 10 s and 60 °C for 30 s. Relative expression levels of *MaCRF* genes were calculated using the 2^−ΔCT^ method.

### 4.7. Statistical Analysis

The datasets consisting of two different groups within the same variety were examined using Tukey’s HSD test. If the letters marked for the two groups of data are completely different, there is a significant difference (usually *p* < 0.05); if they share at least one identical letter, there is no significant difference. *MaEF1* was used as a reference control (Forward primer: CGGAGCGTGAAAGAGGAAT; Reverse primer: ACCAGCTTCAAAACCACCAG). Each experiment included three technical replicates and was performed in three independent biological experiments. The data are presented as the mean ± standard deviation (SD) from three biological replicates.

## 5. Conclusions

In the present study, through comprehensive genomic analysis, eight CRF family genes in banana were identified and characterized. These eight MaCRF genes exhibited tissue-specific expression patterns, indicating diverse functional roles in banana growth and development. Distinct expression profiles between *Foc* TR4-resistant Z8 and susceptible Z1 cultivars for MaCRF4 in root were found, which exhibited strong induction in susceptible Z1, and transient early upregulation followed by downregulation in resistant Z8. In addition, MaCRF3 and MaCRF4 demonstrated significant responsiveness to multiple stresses including osmotic pressure, salt stress, ABA signaling and low temperature treatment. These results establish the MaCRF gene family as crucial regulators of both biotic and abiotic stress responses in banana. This work provides both fundamental insights into banana stress biology and practical targets for developing climate-resilient banana varieties, addressing critical challenges in sustainable tropical agriculture.

## Figures and Tables

**Table 1:** Secondary structure prediction of CRF family proteins in *M. acuminata* DH pahang.

Gene Name	Alpha Helix/%	Extended Strand/%	Beta Turn/%	Random Coil/%	Secondary Structure Element Distribution	Tertiary Structure
*MaCRF1*	31.96	4.12	2.06	61.86	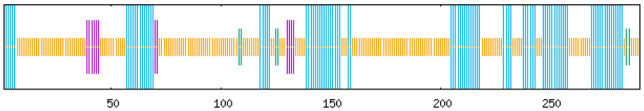	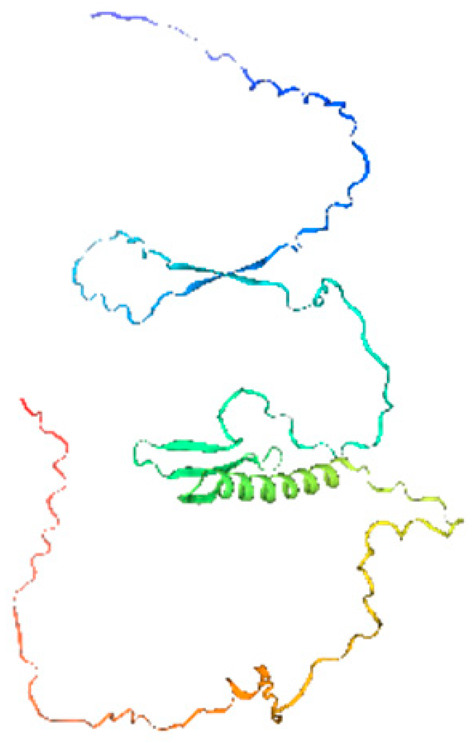
*MaCRF2*	16.00	5.67	1.33	77.00	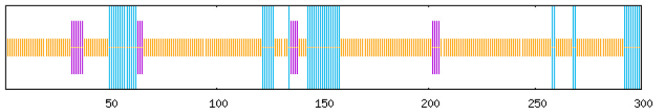	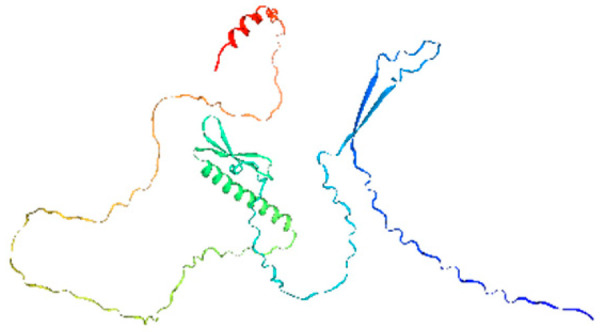
*MaCRF3*	18.85	7.85	2.62	70.68	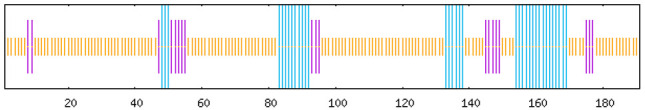	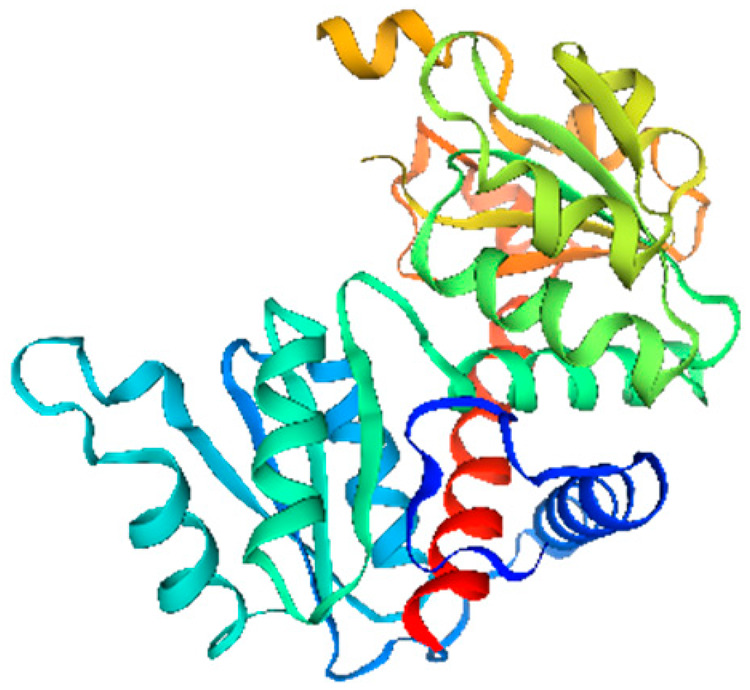
*MaCRF4*	22.70	4.96	1.42	70.92	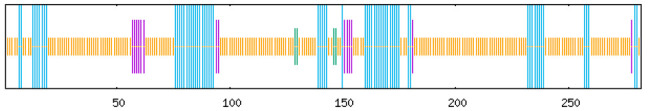	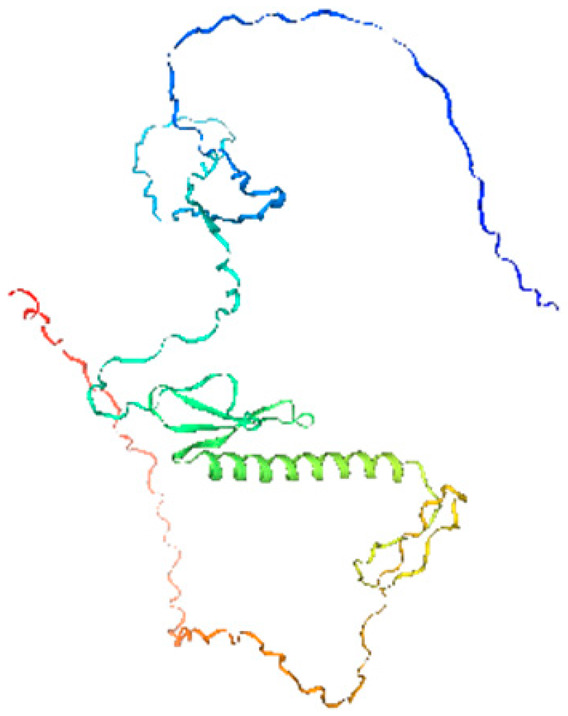
*MaCRF5*	17.14	6.43	1.79	74.64	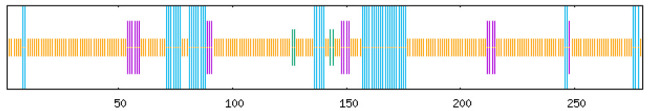	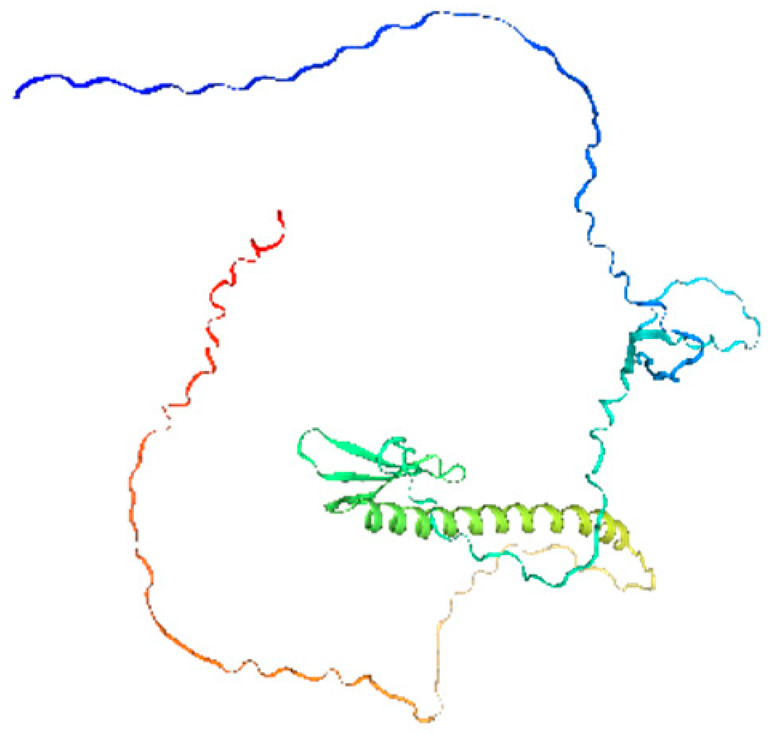
*MaCRF6*	17.42	4.84	1.29	76.45	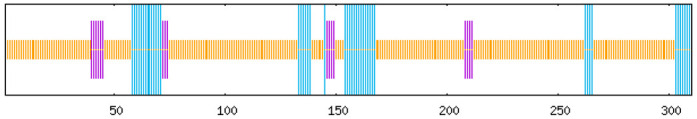	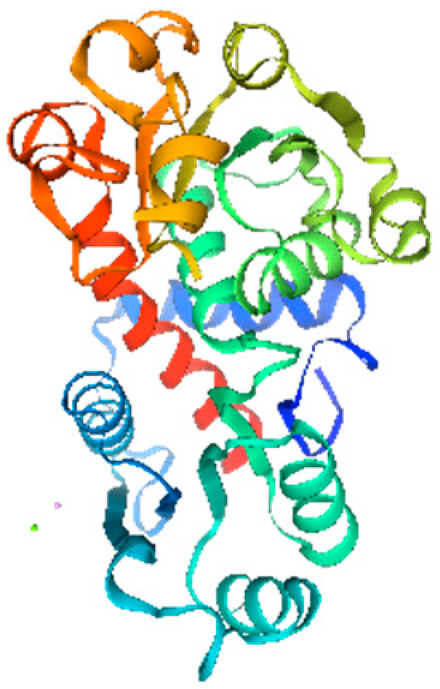
*MaCRF7*	20.07	8.45	1.41	70.07	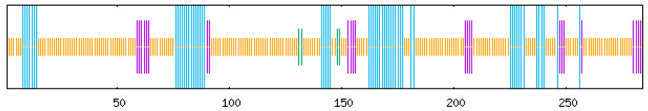	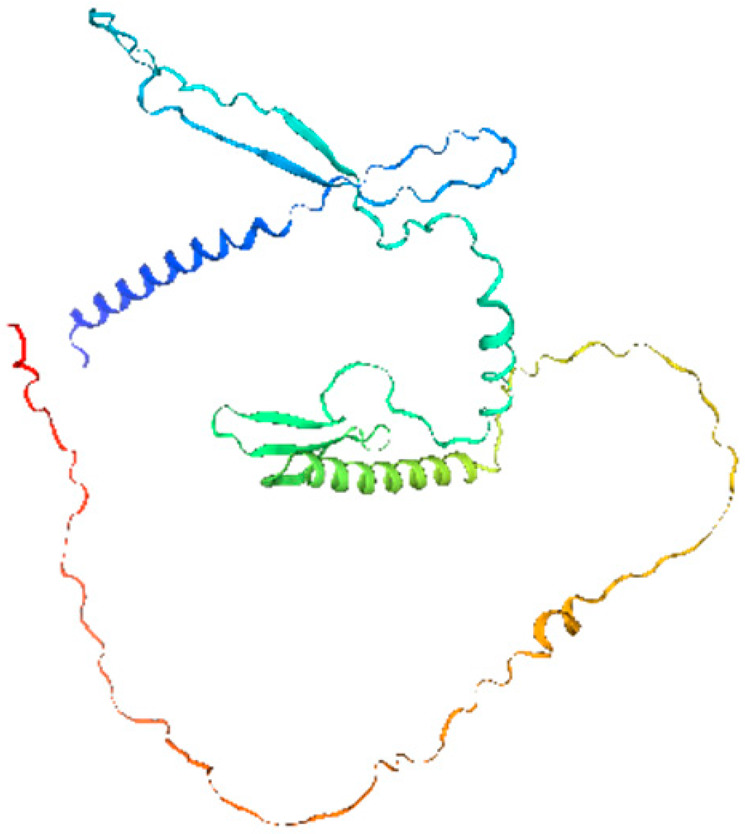
*MaCRF8*	15.64	4.91	1.23	78.22	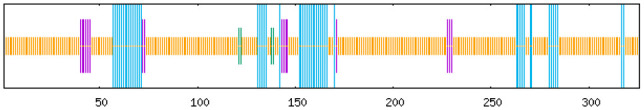	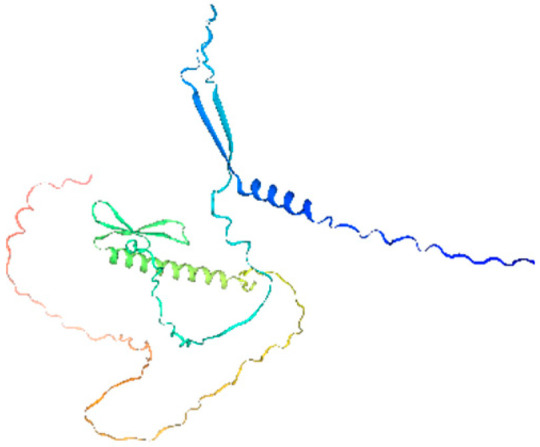

## Data Availability

The original contributions presented in this study are included in the article/Supplementary Materials. Further inquiries can be directed to the corresponding authors.

## Supplementary Materials

The following supporting information can be downloaded at: https://www.mdpi.com/article/10.3390/ijms262311316/s1.
