# Supplementary figures and images for: Genome-Wide Identification of Cytokinin Response Factors (CRFs) Involved in Stress Responses in Banana (Musa acuminata)
Source: Int J Mol Sci. 2025 Nov 23;26(23):11316. doi: 10.3390/ijms262311316 (PMC12692116; doi:10.3390/ijms262311316)

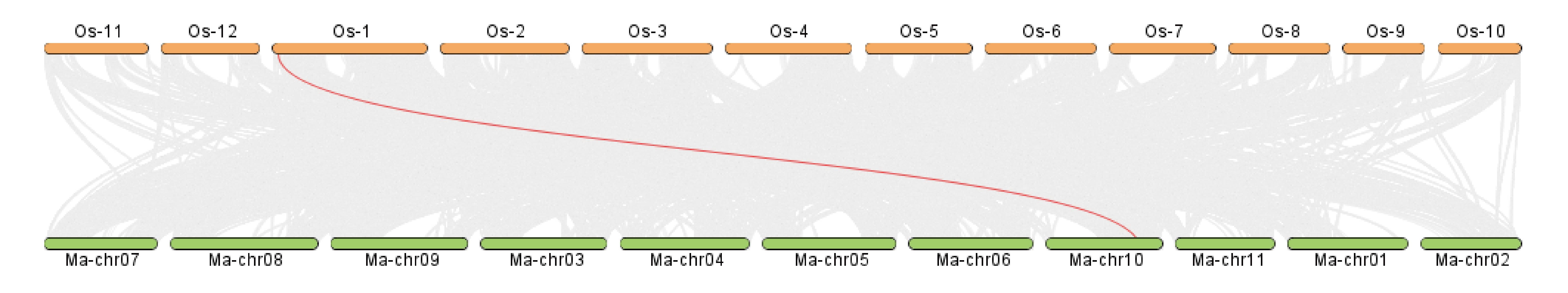

Supplement: Supplementary file 1 [file ijms-26-11316-s001.zip › Figure S2.jpg]
